# Breast cancer related lymphoedema following treatment in the Edinburgh Breast Unit

**DOI:** 10.1016/j.jpra.2026.04.006

**Published:** 2026-04-21

**Authors:** Tim Buick, Ellen Hardie, Josie Cameron, Dhanajay Kulkarni

**Affiliations:** a Department of Plastic and Reconstructive Surgery, St John’s Hospital, Livingston EH54 6PP, Edinburgh, UK; b Edinburgh Breast Unit, Western General Hospital Edinburgh, EH4 2XU, Edinburgh, UK; c Edinburgh Cancer Unit, Western General Hospital, EH4 2XU, Edinburgh, UK

**Keywords:** Lymphoedema, Axillary node clearance, Upper limb, Breast cancer related lymphoedema

## Abstract

There is very little published data on the epidemiology of breast cancer related lymphoedema (BCRL) in the Scottish population. In Scotland, BCRL is treated by a variety of practitioners across the country and there is no national system to audit outcomes. With recent advances in lymphatic surgery and new guidance from NICE, there is a group of patients who could benefit from prophylactic lymphatic surgery at the time of their primary breast cancer excision. In this short communication we review 5 years of referrals to the lymphoedema unit attached to the Edinburgh Breast Unit. We show the rate of upper limb lymphoedema was 14.6% after oncological breast cancer surgery, and patients undergoing sentinel node biopsy and breast conserving surgery had a 3.3% risk of breast oedema. Importantly, during the process of data collection, we noted how patient records are often fragmented across several electronic and paper record systems. There is a clear need here for a national managed audit network which would enable accurate data collection across health boards and could be used to monitor outcomes of lymphoedema therapy and lymphatic surgery. We suggest, to begin with lymphoedema data could be added to the cancer audit network QPI’s (quality performance indicators). This would allow annual assessment of patient outcomes in a rapidly changing surgical field.

## Introduction

There is minimal data on the epidemiology of breast cancer related lymphoedema (BCRL) in the Scottish population. BCRL includes upper limb lymphoedema (ULL) or breast oedema, and can occur following axillary node clearance (ANC) or sentinel node biopsy (SNB) with breast conserving surgery (BCS). In Scotland BCRL is treated by a variety of practitioners across the country and there is no national system to audit outcomes. With recent advances in lymphatic surgery and new guidance from NICE,^1^ there is a group of patients who could benefit from prophylactic lymphatic surgery at the time of their primary breast cancer excision. Evidence shows this can reduce episodes of cellulitis and improve patients’ quality of life.^2^, ^3^ However, selecting a patient with the correct risk profile is key to these procedures being successful.^4^

In this short communication we review 5 years of referrals to the lymphoedema unit attached to the Edinburgh Breast Unit. Primary surgical intervention, adjuvant treatment and comorbidities for each referral was collated from the South East Scotland Cancer Audit Network (SCAN). We hope this lays the groundwork for future collaborative Scottish audit.

## Methods

This retrospective case series included 96 patients referred to a regional lymphoedema service between March 2020–2025. Patients having ANC or SNB with subsequent ULL or breast oedema diagnosis were included. Patients with venous cording, lymphatic cording or oedema in sites other than upper limb/breast were excluded.

Data on lymphoedema site, severity and date of first assessment were collated. The lymphoedema severity at first assessment was recorded by physiotherapists who classified based on excess limb volume as mild (<20%), moderate (20–40%) or severe (>40%). Limb volume was calculated using cylinder-based circumference measurements every 4 cm proximal from the ulnar styloid with the normal arm used as a reference point. Breast oedema was diagnosed clinically as indurated pitting of the skin not associated with erythema or heat. Additional data collected from SCAN included surgery type, date of surgery, tumor type, radiotherapy site, BMI, medications, number of nodes excised, smoking status and age. Additionally, the number of breast operations in our lymphoedema service catchment area (GP postcodes EH1-EH20) was requested from SCAN.

## Results

**Figure 1:**
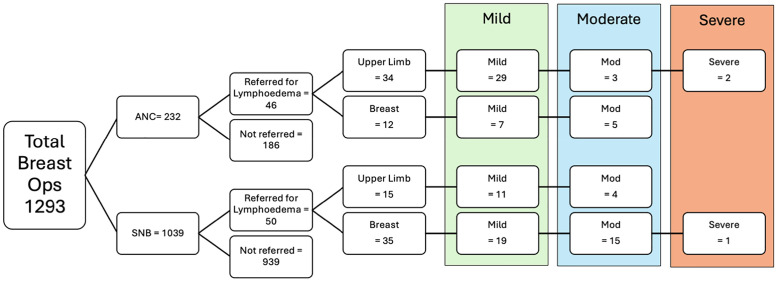
Selection of patients in our study showing those who were referred and those who were not. Upper limb and breast oedema are further categorized by excess limb volume into mild, moderate and severe.

Between March 2020 and 2025, 1293 breast cancer operations were performed in our catchment area. Figure 1 shows the breakdown of upper limb and breast oedema in this group.

The number of patients who developed ULL of any severity following ANC was 34 out of 232 (14.6%). The rate of breast oedema after ANC was 5%. More than 1.5% of patients who had SNB developed ULL, and 3.3% developed breast oedema. Table 1 shows data for patients who had ANC (*N* = 232) and compares key characteristics of those who were referred for ULL and those who were not by lymphoedema severity. Due to low numbers, formal statistical comparisons were not appropriate.

**Table 1:** Comparison of ANC patients not referred to the lymphoedema service (*N* = 232) and ANC patients who were referred with ULL (*N* = 34) which was mild (*N* = 29), moderate (*N* = 3) or severe (*N* = 2).

Axillary node clearance with upper limb lymphoedema	Not referred *n* = 186	All lymph severities *n* = 34	Mild *n* = 29	Moderate *n* = 3	Severe *n* = 2
Mean BMI (kg/m^2^)	27.8	30.6	29.8	35.2	35.6
Mean age (years)	60.9	59.2	58.1	59.3	75.5
Tumor type:					
Patients with Lobular	15%	9%	10%	0%	0%
Patients with NST	73%	88%	86%	100%	100%
Patients with Other	11.9%	3%	3%	0%	0%
Pathology:					
Lymphatic invasion present	43%	44%	41%	66%	50%
No lymphatic invasion present	56%	56%	58%	33%	50%
Average number of nodes excised	14.9	14.4	14.8	11.3	13.5
Average number of invasive nodes	4.2	3.5	3.0	6.7	5.0
Radiotherapy:					
Patients with Short RT (2600cGy/5 fractions)			31%	0%	0%
Patients with Long RT (4005cGy/15 fractions)			68%	100%	100%
Chemotherapy:					
Chemotherapy	74.7%	70.5%	68%	100%	50%
No chemotherapy	25.3%	29.4%	31%	0%	50%
Average Time to assessment (years)			0.9	0.9	0.8

Groups are separated by tumor type, pathology and radiotherapy schedule. Short course radiotherapy included 2600/5 with or without a boost of 1200/4. Long course radiotherapy included 4005/15 with or without a boost of 1200/4.

## Discussion

When comparing lymphoedema and non-lymphoedema groups (Table 1) our results reflect that of published literature.^5^ As expected, patients who acquired moderate to severe lymphoedema were often obese (BMI>30), had a higher age and had been treated with long course radiotherapy.

Even though the rate of breast oedema following SNB and breast conserving surgery was low (3.3%), this contributed to 36% of the workload for the lymphoedema service. Breast conserving surgery involves more dissection of the breast architecture and so one would expect an interruption to lymphatics. It is not surprising then that patients develop isolated breast oedema.

The rate of ULL following ANC in this study was 14.6%; this is lower than published rates of 20%^5^ and there are two broad reasons for this. Firstly, it is possible there is selection bias in our patient sample population. Some patients in our region may have seen another service or an independent practitioner such is the fragmented nature of lymphoedema services in Scotland. Secondly, the average time to developing ULL symptoms was 10 months (Table 1) and so patients who were operated on at the end of our study period may not have developed symptoms yet.

As this is a retrospective study, there was no prior sample size/power calculation. In addition, since no formal statistical testing or measures of statistical significance are reported, the current data do not allow a robust conclusion that the number of nodes excised, tumor type, lymphovascular invasion, or chemotherapy do not influence subsequent ULL development. As such, these variables should remain candidates for inclusion in future, adequately powered analyses to determine their true association with ULL risk.

## Conclusion

To our knowledge this is the first epidemiological study of BCRL in our region. Importantly, during the process of data collection, we noted how patient records are often fragmented across several electronic and paper record systems. There is a clear need here for a national managed audit network which would enable accurate data collection across health boards and could be used to monitor outcomes of lymphoedema therapy and lymphatic surgery. We suggest, to begin with lymphoedema data could be added to the cancer audit network QPI’s (quality performance indicators). This would allow annual assessment of patient outcomes in a rapidly changing surgical field.

## Declaration of competing interest

None declared.

## References

[1] NICE Guideline NG101 (2025).

[2] Cho M.J., Senger J.L., Park K.U., Hansotia K., Chratian S., Kadle R. (2025). Preventing breast cancer-related lymphedema: a comprehensive analysis of a 9-year single-center experience of prophylactic lymphovenous bypass. Ann Surg Oncol.

[3] Thomas M., Pike C., Humphreys I., Bragg T., Ghattaura A. (2023). Impact and outcomes after lymphaticovenous anastomosis for 150 cases of lymphoedema followed up over 24 months. J Plast Reconstr Aesthet Surg.

[4] Sierla R., Dylke E., Poon S., Shaw T., Kilbreath S. (2025). Attaining consensus on a core dataset for upper limb lymphoedema using the Delphi method: a foundational step in creating a clinical support system. Health Inf Manag.

[5] DiSipio T., Rye S., Newman B., Hayes S. (2013). Incidence of unilateral arm lymphoedema after breast cancer: a systematic review and meta-analysis. Lancet Oncol.

